# Racial and ethnic differences in the degree of participation and retention in a decentralized cohort study of COVID-19 immunization in patients with inflammatory bowel diseases

**DOI:** 10.1017/cts.2024.5

**Published:** 2024-01-18

**Authors:** Margie Boccieri, Riley Craig, Xian Zhang, Ann M. Firestine, Millie D. Long, Michael D. Kappelman

**Affiliations:** 1 Pediatric Gastroenterology, The University of North Carolina at Chapel Hill School of Medicine, Chapel Hill, NC, USA; 2 Gastroenterology, The University of North Carolina at Chapel Hill School of Medicine, Chapel Hill, NC, USA

**Keywords:** Decentralized research, diversity equity inclusivity, inflammatory bowel diseases, COVID-19, virtual study

## Abstract

**Introduction:**

Disparities in the recruitment of minority populations in research are well-documented. However, the degree of participation and retention of minorities following enrollment is less known, particularly in decentralized studies. Although decentralized clinical research methods may allow researchers to engage broader study populations with less participation burden, they may present different retention challenges. To evaluate racial and ethnic differences in the degree of participation after enrollment in a decentralized study, we analyzed data from a cohort of patients with inflammatory bowel diseases following COVID-19 immunization.

**Methods:**

We compared by race and ethnicity the following post-enrollment participation metrics: response to > 50% of follow-up surveys, donation of a blood sample for antibody testing, consent to use of bio samples for future research, and withdrawal prior to study completion.

**Results:**

Overall, we observed higher levels of post-enrollment study participation among non-Hispanic White (NHW) participants as compared to Black or Hispanic participants: 95% of NHW participants completed follow-up versus 87% of Black participants and 91% of Hispanic participants, 73% of NHW participants provided bio samples versus 64% Black participants and 67% Hispanic participants, and 65% of NHW participants provided consent for future research versus 62% of Black participants and 52% of Hispanic participants.

**Conclusions:**

Our findings demonstrate that the degree of study participation after enrollment in this decentralized study differed by race and ethnicity, indicating that attention to diversity, equity, and inclusion is needed not only in clinical research recruitment but also throughout study administration.

## Introduction

Conducting inclusive research is particularly important for chronic conditions such as IBD, which impact more than 1.5 million Americans of all races and ethnicities [1–3]. Emerging research suggests that Black and Hispanic patients experience disparities in IBD care and outcomes [4]. As these populations are also underrepresented in IBD research [5,6], increasing the representativeness of research in this sector may be an important driver of health equity.

Catalyzed by recent technological innovations and the COVID-19 pandemic, decentralized clinical research studies recruit, screen, collect data, and deliver interventions directly to patients, where they live and work, rather than operating through clinical trial sites as intermediaries [7]. By reducing common barriers to research participation including, geographic distance, travel time, and scheduling constraints, decentralization may improve research accessibility to historically underrepresented groups. However, this has not been rigorously examined and remote clinical research could potentially have the opposite effect – perpetuating and exacerbating inequities. Indeed, populations historically underrepresented in research may have lower technology uptake [8]. Moreover, the decreased personalized connection between researchers and potential study participants may make it harder to overcome other barriers to research participation such as mistrust, low research awareness, and other cultural concerns.

Recruitment is only the first step in conducting inclusive, equitable research. Ensuring robust participation and retention after enrollment is even more important. Prior research in traditional study settings has indicated that degree of participation and retention following enrollment differs by race and ethnicity, and several mitigating strategies have been proposed including use of higher levels of personal and continuing contact and leveraging community health workers [9–11]. However, post-enrollment participation and retention have not been well-studied in decentralized research studies, where personal contact is more difficult to provide.

Partnership to report effectiveness of vaccination in populations excluded from initial trials of COVID (PREVENT COVID) is a fully decentralized observational study to evaluate the effectiveness and safety of COVID-19 vaccines in patients with IBD, a population frequently treated with immune suppression that may attenuate vaccine response. As IBD patients treated with immune suppression were excluded from the initial clinical trials of COVID-19 vaccines, understanding vaccine effectiveness and safety in this population was recognized as a critical research need [12]. Participants were followed for 18 months through online surveys. Participants had the option to undergo blood collection to test for IgG antibodies specific to SARS-CoV-2 at a LabCorp facility in their community, and could consent for long-term biobanking of leftover samples. Thus, this study provided a unique opportunity to evaluate racial and ethnic differences in the degree of study participation and retention following enrollment in a decentralized research study of a high-priority topic (COVID-19 immunization) in a population affected by a complex, chronic condition.

## Methods

### Study setting

Recruitment for the PREVENT COVID study was conducted primarily through two organizations with large constituencies in our target population: the Crohn’s & Colitis Foundation and IBD Partners. IBD Partners is a patient-powered research network of more than 12,000 members run by two of the study’s investigators. These organizations publicized the study through their listservs and through social media posts on Facebook, Twitter, and Instagram. Study investigators also conducted information sessions on Facebook Live. All interested participants were referred directly to the study website to enroll. We did not collect data on a participant’s referral method. All data was collected and stored on secure servers at the University of North Carolina, Chapel Hill. Individuals self-screened at enrollment attesting that they: had been diagnosed with IBD, had received their first COVID-19 vaccine within the proceeding 90 days, lived in the United States, had access to the internet to complete regular study surveys, were willing to remain in the study for 18-months, and were willing to answer survey questions in English. Due to time and resource constraints, study materials were not available in any language other than English. After successful self-screening, participants provided electronic informed consent and completed a baseline survey to collect two forms of contact information, demographic and disease characteristics, and detailed immunization data. Study personnel analyzed the immunization data provided to ensure patients met the eligibility requirements. For example, if a participant enrolled outside of the 90-day window for an initial COVID-19 vaccine, they were withdrawn and considered a screen fail.

Baseline and follow-up surveys at 1 month, 2 months, 6 months, 9 months, 12 months, 15 months, and 18 months following enrollment ascertained vaccine type, date, side effects, changes in IBD disease course, and COVID-19 infections. These surveys were accessed through a link emailed to the participant. Each survey remained open for 21 days during which participants were sent up to 3 automated reminders. After 14 days of non-response, study staff attempted to reach participants by phone and personal emails to remind them to complete the surveys. When surveys were returned as undeliverable, study personnel attempted to contact the participant and the participant’s backup contact to update email contact information. This happened rarely and the instances were not tracked. To further promote participant engagement and encourage retention, we also sent regular newsletters publicizing preliminary results and created a study website for return of aggregate results.

In addition to survey-based follow-up, participants were invited to provide up to three blood samples to measure IgG antibodies specific to SARS-CoV-2. These blood samples were collected approximately 3 months after a participant’s first COVID-19 vaccine, 1215 months after their first COVID-19 vaccine, and 3-8 weeks following their first booster vaccine. Participants who agreed to antibody testing also had the opportunity to consent to long-term storage of their bio samples for future research use.

All data were self-reported and not verified through the patient’s IBD clinician.

Participants were compensated for completion of baseline and follow-up surveys and for providing blood samples. Compensation was paid at one time at the conclusion of the study. IgG antibody test results were returned to participants as an additional incentive for participation.

PREVENT COVID opened enrollment on March 15, 2021. The planned sample size was 1,500 participants set for budgetary reasons and to provide adequate power for analysis. Due to rapid accrual and the desire to learn as much as possible, the sponsor provided supplemental funding to enroll additional participants. Within 10 weeks, the study exceeded by double its initial enrollment target. After enrolling more than 3,000 participants, the study was closed to new adult enrollment on May 31, 2021.

Results of safety, immunogenicity, and efficacy analyses have been previously published [13–16]. In this report, we focus on analyzing participation and retention by race and ethnicity.

### Analysis

The primary exposures were self-reported race and ethnicity. Study outcomes included the proportion of adult patients who (1) responded to > 50% of follow-up surveys, (2) withdrew or were lost to follow-up prior to study completion, (3) provided a blood sample for antibody testing, and (4) consented to long term storage and future use of bio samples. Bivariate comparisons were conducted using the chi-square test or Fisher’s exact test. A *p* < 0.05 was considered statistically significant. The study protocol was approved by the University of North Carolina at Chapel Hill Institutional Review Board.

## Results

### Study population and data collected

Demographic and disease characteristics of the study population are provided in Table 1. The ability to complete surveys over the Internet was one of the enrollment requirements for the study, so we did not choose to assess whether our study population might have had easier or more difficult access to the Internet based on where they lived.

**Table 1.** Characteristics of PREVENT COVID adult population

		Race									Ethnicity		
	Total	Asian	Black/ African American	More than one race	Native American or Other	White	Overall *p* value	White vs. Black *p* value	Non-Hispanic	Hispanic	Unknown	Overall *p* value	Hispanic vs. Non-Hispanic *p* value
**Total participants**	3210	58 (1.8%)	39 (1.2%)	53 (1.7%)	39 (1.2%)	3021 (94.1%)			3098 (96.5%)	101 (3.1%)	11 (0.03%)		
**Demographics**													
**Gender**													
Female	2315 (72.1%)	31 (1.3%)	32 (1.4%)	34 (1.5%)	23 (1.0%)	2195 (94.8%)			2236 (96.6%)	70 (3.0%)	9 (0.04%)		
Male	876 (27.3%)	27 (3.1%)	7 (0.8%)	19 (2.2%)	16 (1.8%)	807 (92.1%)			843 (96.2%)	31 (3.5%)	2 (0.2%)		
Other	19 (0.6%)					19 (100%)			19 (100%)				
**Age**													
Age (mean)	43.4												
18–24	297 (9.3%)	8 (2.7%)	4 (1.3%)	15 (5.1%)	8 (2.7%)	262 (88.2%)			271 (91.2%)	24 (8.1%)	2 (0.7%)		
25–34	714 (22.2%)	16 (2.2%)	9 (1.3%)	13 (1.8%)	8 (1.1%)	668 (93.6%)			683 (95.7%)	30 (4.2%)	1 (0.1%)		
35–44	843 (26.3%)	18 (2.1%)	9 (1.1%)	20 (2.4%)	13 (1.5%)	783 (92.9%)			816 (96.8%)	23 (2.7%)	4 (0.5%)		
45–54	573 (17.9%)	8 (1.4%)	11 (1.9%)	4 (0.7%)	7 (1.2%)	543 (94.8%)			552 (96.3%)	19 (3.3%)	2 (0.3%)		
55–64	436 (13.6%)	6 (1.4%)	4 (0.9%)	1 (0.2%)	3 (0.7%)	422 (96.8%)			430 (98.6%)	4 (0.9%)	2 (0.5%)		
65–older	347 (10.8%)	2 (0.6%)	2 (0.6%)			343 (98.8%)			346 (99.7%)	1 (0.3%)			
**US region**													
Midwest	783 (24.4%)	9 (1.1%)	5 (0.6%)	10 (1.3%)	5 (0.6%)	754 (96.3%)			766 (97.8%)	14 (1.8%)	3 (0.4%)		
Northeast	759 (23.6%)	7 (0.9%)	9 (1.2%)	7 (0.9%)	8 (1.1%)	728 (95.9%)			741 (97.6%)	16 (2.1%)	2 (0.3%)		
South	970 (30.2%)	17 (1.8%)	20 (2.1%)	15 (1.5%)	10 (1.0%)	908 (93.6%)			923 (95.2%)	45 (4.6%)	2 (0.2%)		
West	698 (21.7%)	25 (3.6%)	5 (0.7%)	21 (3.0%)	16 (2.3%)	631 (90.4%)			668 (95.7%)	26 (3.7%)	4 (0.6%)		
**Diagnosis**													
Crohn’s Disease	2147 (66.9%)	26 (1.2%)	29 (1.4%)	32 (1.5%)	22 (1.0%)	2038 (94.9%)			2082 (97%)	59 (2.7%)	6 (0.03%)		
Ulcerative Colitis	965 (30.1%)	31 (3.2%)	10 (1.0%)	18 (1.9%)	16 (1.7%)	890 (92.2%)			922 (95.5%)	38 (3.9%)	5 (0.5%)		
Indeterminate Colitis/ IBD-unspecified	60 (1.9%)			1 (1.7%)		59 (98.3%)			58 (96.7%)	2 (3.3%)			
Missing	38 (1.6%)	1 (2.6%)		2 (3.5%)	1 (2.6%)	34 (89.5%)			36 (94.7%)	2 (5.3%)			
**Study participation**													
Responded to>50% of surveys	2982 (92.9%)	51 (88.0%)	32 (82.1%)	48 (90.6%)	35 (89.7%)	2816 (93.2%)	0.031	0.006	2881 (93.0%)	90 (89.1%)	11 (100%)	0.214	0.135
Completed at least one blood draw	2327 (72.5%)	39 (67.2%)	25 (64.1%)	36 (67.9%)	24 (61.5%)	2203 (72.9%)	0.252	0.219	2250 (72.6)	68 (67.3%)	9 (81.8%)	0.395	0.241
Agreed to Biobanking	2061 (64.2%)	29 (50.0%)	24 (61.5%)	28 (52.8%)	19 (48.7%)	1961 (64.9%)	0.012	0.661	1999 (64.5%)	53 (52.5%)	9 (81.8%)	0.022	0.013
Withdrawn	174 (54.2%)	3 (5.2%)	5 (12.8%)	5 (9.4%)	3 (7.7%)	158 (5.2%)	0.168	0.036	165 (5.3%)	9 (8.9%)	0 (0.0%)	0.214	0.118

Recruitment in the PREVENT COVID study was robust and vastly surpassed our expectations. Between March 17 and May 31, 2021, 3,594 adults expressed interest in enrolling. Of this number, 31 were screen fails and 353 (10%) did not follow through with all enrollment requirements and were excluded. We ultimately enrolled 3,210 adults from 49 U.S. States, Puerto Rico, and the District of Columbia. Over the course of the study, 174 participants withdrew before study completion. Reasons for early withdrawal were tracked, but no formal analysis was done on when in the 18-month follow-up period participants withdrew. However, the vast majority of withdrawn individuals were marked Lost to Follow-Up after they had not completed three consecutive surveys and did not respond to attempts by study staff to reach them, or their backup contact, by email or telephone. Most individuals marked Lost to Follow-Up were withdrawn between 9- to 15-months. It was rare for emails to bounce back or for phone numbers to have been disconnected; in most instances, the participant simply did not respond to emails or phone messages.

Overall follow-up and study participation was robust. More than 91% of participants completed all required questions of the first four follow-up surveys (at 1 month, 2 months, 6 months, and 9 months after enrollment) and more than 87% completed all required questions in the last three surveys at 12, 15 and 18-months.

PREVENT COVID also collected more than 5,800 voluntary blood samples, with 73% of participants providing at least 1 sample.

### Participation by race and ethnicity

**Figure 1.**
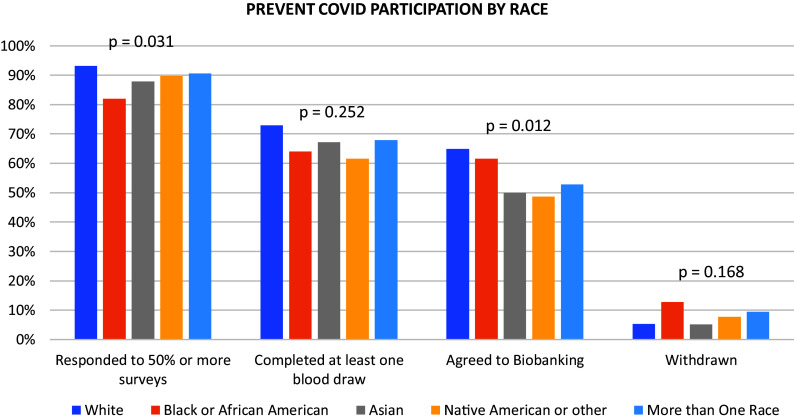
Prevent COVID participation by race.

**Figure 2.**
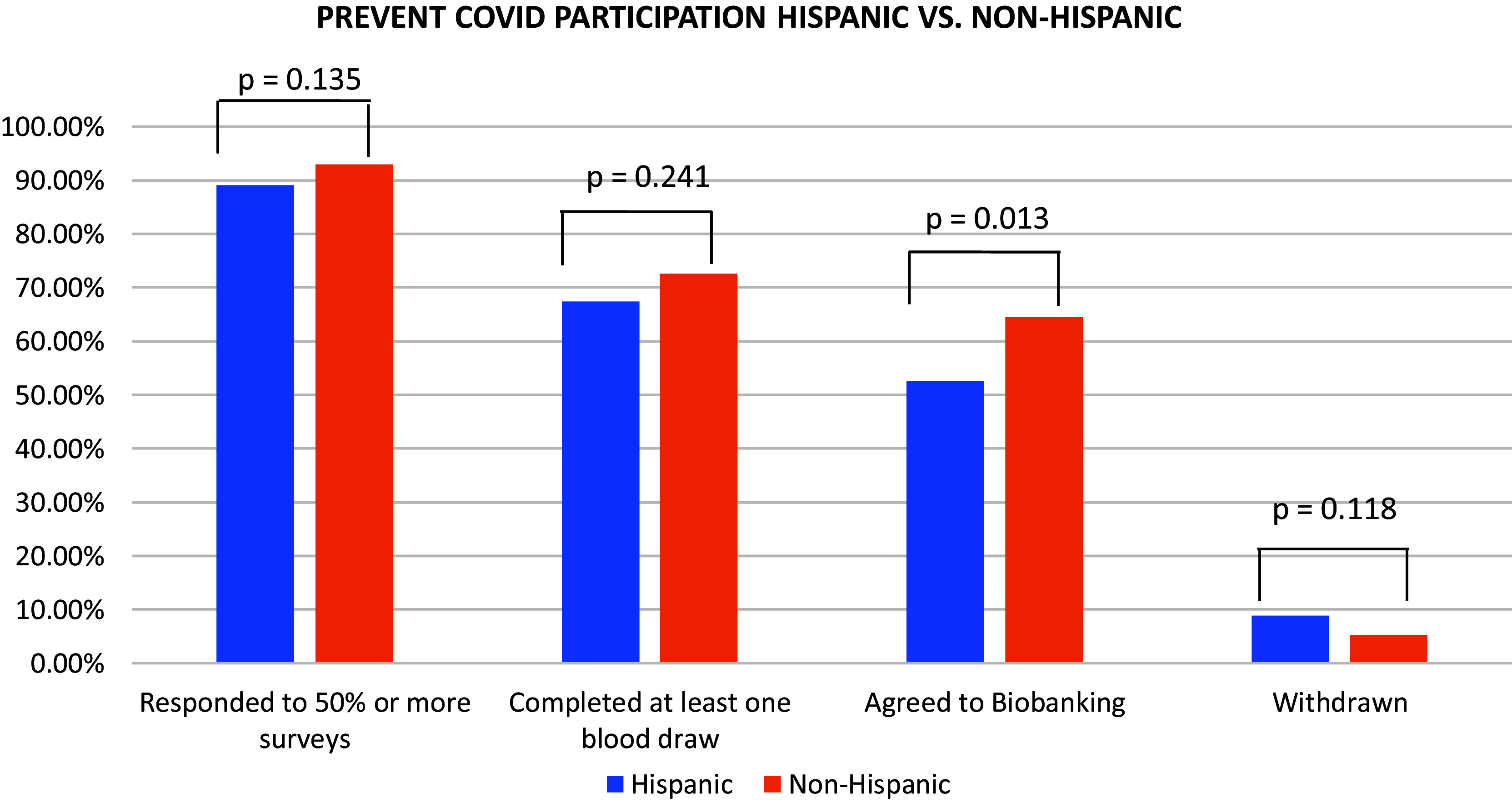
Prevent COVID participation Hispanic vs. non-Hispanic.

For all participation measures, non-White and Hispanic participants completed numerically fewer study components and were more likely to withdraw before study completion than their White and non-Hispanic counterparts (Table 1, Figs. 1, 2).

The proportion of participants responding to > 50% of surveys differed significantly among racial groups (*p* = 0.031 Table 1, Fig. 1) and when specifically comparing White and Black/African American participants (93% vs 82%, *p* = 0.006).

Similarly, compared to White participants, a higher proportion of Black/African American participants were lost to follow-up or withdrew early (13% vs 5%, *p* = 0.036 Table 1). Early withdrawal rates were also higher among Hispanic participants (9%) when compared to non-Hispanic participants (5%) but this difference was not statistically significant (*p* = 0.118) (Table 1, Fig. 2).

A trend toward greater participation in antibody testing among Whites (73%) and non-Hispanics (73%) compared to other racial and ethnic groups (Black/African American, 64%, Asian, 67%, Native American or Other, 62%, More than One Race 68%, Hispanic, 67%) was not statistically significant (Fig. 1, Fig. 2).

We also observed significant differences across racial groups in the proportion of participants consenting to biobanking and future research use of blood samples (White, 65%, Black/African American, 62%, Asian, 50%, Native American or Other, 49%, More than One Race 53%) (*p* = 0.012). Additionally, non-Hispanic participants (65%) were more likely than Hispanic participants (53%) to consent to biobanking (*p* = 0.013) (Fig. 2).

## Discussion

Among participants enrolled in this decentralized study of COVID-19 vaccine effectiveness and safety, we observed high levels of survey completion, little attrition, and a high rates of blood collection and consent for biobanking, supporting the feasibility of fully remote, prospective cohort studies. Importantly, we noted higher follow-up survey participation and overall retention among White and non-Hispanic participants compared to those of other racial and ethnic groups. Similarly, White and non-Hispanic participants were more likely to consent to biobanking for potential future research. Given the growing emphasis on research inclusivity and the increasing trend toward decentralized clinical research, these findings are particularly timely and relevant highlighting the need to develop strategies to promote equitable study participation and retention following enrollment.

Our study adds to the limited research in the field. An evaluation of 24 traditional trials conducted by the National Drug Abuse Treatment Clinical Trials Network demonstrated no statistical differences in retention across racial and ethnic groups [17]. However, a more recent analysis of retention in a decentralized study demonstrated higher retention of non-Hispanic whites as compared to Black and Hispanic participants [18] consistent with our study’s findings. Taken together, this emerging literature regarding participant engagement and retention in decentralized studies suggests the need to develop and test targeted strategies to promote engagement and retention of minority study participants. As an initial step, qualitative research will be required to better understand potential barriers to continued study participation among different racial/ethnic minority groups and identify mitigating strategies. Consistent with other researchers [19], we acknowledge that access to electronic devices, reliable internet, and user comfort completing web-based surveys, along with cultural issues, language barriers, and/or participants’ attitudes and trust toward healthcare, in general, may have contributed to reduced long-term participation in our cohort. Specific to PREVENT COVID, participation in antibody testing through LabCorp required transportation and the flexibility to make an appointment during business hours.

Our overall study population skewed toward non-Hispanic White (NHW) enrollment. Given the pressing need to generate data quickly, we prioritized overall recruitment speed over diversity. Our referral sources, the Crohn’s & Colitis Foundation and the IBD Partners cohort, are comprised of largely White, non-Hispanic constituents. Furthermore, IBD affects nearly twice as many NHWs as compared to minority groups [20]. Additionally, we closed cohort enrollment within several months of the initial vaccine EUA, so vaccine hesitancy in racial and ethnic minorities also impacted enrollment [21]. Lastly, due to time and financial restraints, we were not able to offer this study in any language other than English which excluded participation from individuals who could not respond to surveys in English. If we were able to offer all study components in Spanish, we might have had greater participation from Hispanic IBD patients. Thus, our study was not designed to evaluate the impact of decentralized study designs on recruitment of underrepresented minority populations.

In summary, our findings demonstrate that the degree of study participation after enrollment in this decentralized study differed by race and ethnicity, indicating that attention to diversity, equity, and inclusion is needed not only in clinical research recruitment but also throughout study administration.
